# Calcium-alginate beads as a formulation for the application of entomopathogenic nematodes to control rootworms

**DOI:** 10.1007/s10340-021-01349-4

**Published:** 2021-02-26

**Authors:** Jinwon Kim, Ivan Hiltpold, Geoffrey Jaffuel, Ilham Sbaiti, Bruce E. Hibbard, Ted C. J. Turlings

**Affiliations:** 1 FARCE Laboratory, Institute of Biology, University of Neuchâtel, Neuchâtel, Switzerland; 2 Seoul Viosys Co. Ltd., Ansan, Gyeonggi-do Republic of Korea; 3 Division of Plant Sciences, University of Missouri, Columbia, MO USA; 4 Agroscope, Route de Duillier 50, P.O. Box 1012, 1260 Nyon 1, Switzerland; 5 USDA-ARS, Plant Genetics Research Unit, University of Missouri, Columbia, MO USA

**Keywords:** Alginate beads, Entomopathogenic nematodes, Maize, Rootworms, Root pest control, Glycerol-induced quiescence

## Abstract

Entomopathogenic nematodes (EPN) have great potential as biological control agents against root-feeding insects. They have a rapid and long-lasting mode of action, minimal adverse effects on the environment and can be readily mass-produced. However, they have a relatively short shelf-life and are susceptible to desiccation and UV light. These shortcomings may be overcome by encapsulating EPN in Ca^2+^-alginate hydrogels, which have been shown to provide a humid and UV protective shelter. Yet, current Ca^2+^-alginate formulations do not keep EPN vigorous and infectious for a prolonged period of time and do not allow for their controlled release upon application. Here, we introduce solid Ca^2+^-alginate beads which we supplemented with glycerol to better retain the EPN during storage and to ensure a steady release when applied in soil. Glycerol-induced metabolic arrest in EPN (*Heterorhabditis bacteriophora*) resulting in quiescence and total retainment of EPN when added to beads made with 0.5% sodium alginate and 2% CaCl_2_·2H_2_O solutions. More than 4,000 EPN could be embedded in a single 4–5-mm diameter bead, and quiescence could be broken by adding water, after which the EPN readily emerged from the beads. In a field trial, the EPN beads were as effective in reducing root damage by the western corn rootworm (WCR, *Diabrotica virgifera virgifera*) as EPN that were applied in water. Although further improvements are desirable, we conclude that Ca^2+^-alginate beads can provide an effective and practical way to apply EPN for the control of WCR larvae.

**Supplementary information:**

The online version contains supplementary material available at 10.1007/s10340-021-01349-4.

## Key Message

- The full potential of entomopathogenic nematodes (EPN) as biological control agents has yet to be achieved.
- Main obstacles include short shelf-life, susceptibility to desiccation and UV light, and untimely release.
- We formulated an alginate bead supplemented with glycerol to induce EPN quiescence.
- The beads can retain more than 4,000 nematodes each, and quiescence can readily be broken by adding water.
- In a field trial, EPN beads effectively reduced root damage by the western corn rootworm.

## Introduction

Entomopathogenic nematodes (EPN) have been shown to be highly effective biological control agents with unique advantages that chemical and other biological pesticides do not have. EPN can kill insect pests within days after infection; tens to hundreds of thousands of new EPN may be produced from a single infested insect host and travel to infect new hosts, thus theoretically allowing them to persist in the field throughout the growing season (Kurtz et al. 2007; Lewis et al. 2006). Moreover, EPN are compatible and can be co-applied with other biological pesticides and agrochemicals (Grewal 2002; Imperiali et al. 2017; Rovesti and Deseö 1990; Shapiro-Ilan et al. 2012; Shapiro-Ilan et al. 2004) and have minimal undesirable effects such as soil contamination, bioaccumulation or adverse effects on non-target organisms (Bathon 1996; Lewis et al. 2006; Peters 1996). Despite these advantages, EPN application in large-scale farming is still rare because of some critical obstacles such as higher production cost as compared to synthetic chemical pesticides and the vulnerability of EPN to environmental factors (Georgis et al. 2006).

The free-living, foraging infectious juveniles (IJs) do not feed and use their stored energy reserves until they find an insect host. Therefore, the viability and infectivity of IJs decrease over time as energy reserves are depleted. This limits the shelf-life of EPN products to maximally a few months even when properly stored (Grewal 2002; Koppenhöfer 2007; Shapiro-Ilan et al. 2006). They are also highly susceptible to UV radiation, heat and desiccation; therefore, many EPN die soon after application due to exposure to air and sunlight, and only a tiny fraction will reach target hosts (Gaugler et al. 1997). In addition, they are mostly applied in liquid formulations that require proper agitation in storage tanks to prevent the EPN from settling down at the bottom (Shapiro-Ilan et al. 2012).

Considerable research effort has gone into the development of suitable EPN formulations with the aim to prolong storage, easy transport and handling, efficient application and enhanced post-application survival (Cruz-Martínez et al. 2017; Grewal 2002; Shapiro-Ilan et al. 2006). For instance, water-dispersible granules have been made of mixtures of clays, silica, cellulose, lignin and starch. When mixed with EPN, the granules cause desiccation stress, so-called anhydrobiosis, and thereby induce quiescence in EPN, which significantly reduces metabolic rate and prolongs EPN longevity with minimum loss of infectivity even at room temperature (Grewal 2000a, 2000b; Matadamas-Ortiz et al. 2014). Another promising formulation strategy is the direct application of EPN-infested insect cadavers, which can be buried directly in soil. Most fresh and infectious EPN are released from the cadavers into the rhizosphere just a few days after application (Del Valle et al. 2008; Jansson et al. 1993). EPN-infested cadavers, however, are fragile and may rupture during transport and handling. Potting soil pre-mixed with EPN-infested cadavers and a tape-formulation of dry cadavers have been developed to improve handling, but still suffer from limited shelf-life and reduced infectivity of EPN, respectively (Deol et al. 2011; Zhu et al. 2011).

Alginate, a comparatively cheap linear polymer comprising continuous or alternating 1 → 4 linked units of β-D-mannuronic acid and α-L-guluronic acid, readily cross-links with divalent cations such as Ca^2+^ to form stable hydrogels (Donati et al. 2005; King 1982). Due to their hydrophilic, biocompatible and biodegradable characteristics alginate gels have been widely studied to be used as a formulation to deliver drugs, viable cells or microorganisms for medical purposes (Dong et al. 2013; Hoffman 2012; Tønnesen and Karlsen 2002), biofertilizer (i.e., *Rhizobium*) and biomicrobial pesticides (Bashan 1998; John et al. 2011; Schoebitz et al. 2013). Alginate hydrogels also show promise for EPN formulation as they provide protection from desiccation, UV light and natural enemies. Several reports describe Ca^2+^-alginate-based EPN formulations (Hiltpold et al. 2012; Kaya et al. 1987; Kaya and Nelsen 1985; Kim et al. 2015; Navon et al. 1998, 2002; Renn 1995), but further improvements are needed to ensure EPN viability and infectivity and to control the release of the nematodes (Kary et al. 2018).

**Fig. 1:**
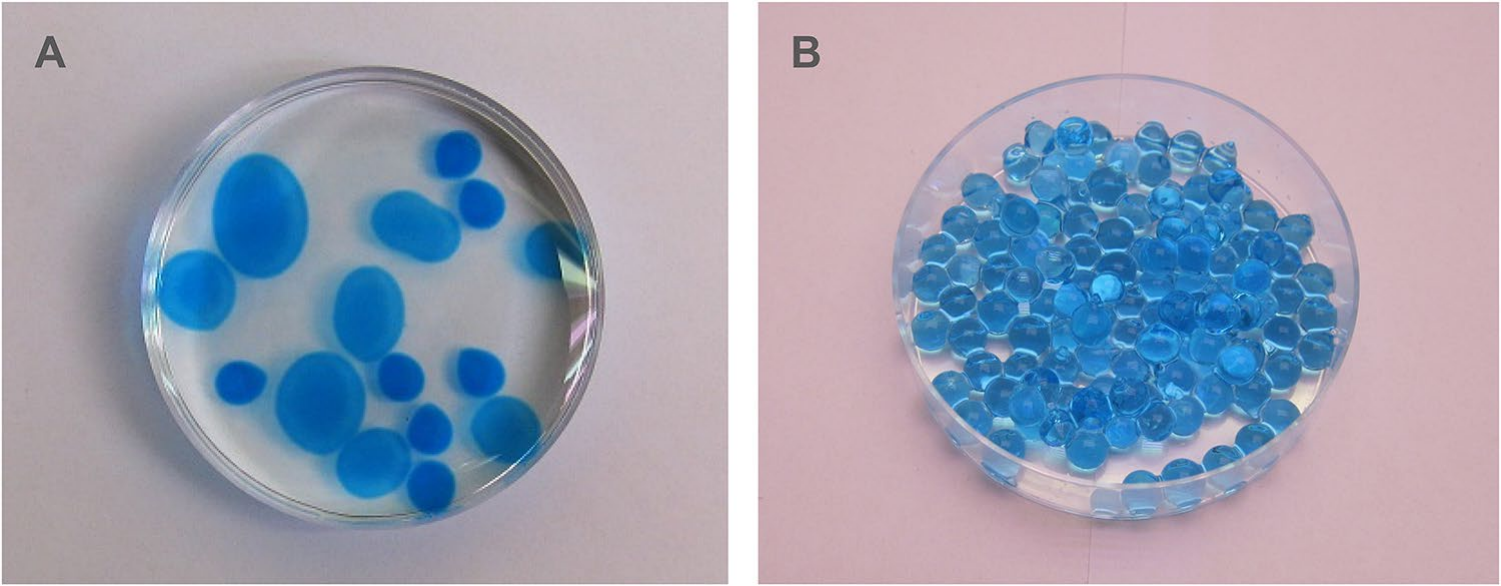
Ca^2+^-alginate capsules **a** and beads **b** inside a petri dish (diam 60 mm). The capsules are of different sizes because they were made with different numbers of droplets of a CaCl_2_∙2H_2_O solution applied to a sodium alginate solution (Kim et al. 2015). Note that each capsule shell surrounds a liquid core. In contrast, the Ca^2+^-alginate beads were solid and were produced by dropping single droplets of sodium alginate solution into a CaCl_2_·2H_2_O solution. All beads are of the same size because for each drop cross-linking occurs immediately due to excessive Ca^2+^ ions, preventing the formation of a liquid core. Figure 1a was reused from Kim et al. 2015 with permission of the editor

We previously showed that producing of Ca^2+^-alginate EPN capsules (or “hollow beads” as defined by Vemmer and Patel (2013)) (Fig. 1) at 4 °C significantly enhanced retainment of EPN as compared to capsules produced at room temperature (Kim et al. 2015). However, a considerable number of encapsulated EPN still escaped from the capsules during a 7-day storage period at room temperature and their viability gradually decreased over time probably because the alginate polymer acts as an oxygen barrier (Sabra et al. 2000). Furthermore, the release of EPN from the capsules after application in the soil was poor.

Here, we present an improved version of an alginate-based EPN formulation. We used alginate beads (“solid spheres” as defined by Vemmer and Patel (2013)) instead of capsules (Fig. 1). Previously reported EPN beads produced from 2% alginate solution dropped into CaCl_2_·2H_2_O or CaCO_3_ solution were found not to be practical because EPN were not able to escape unless the beads were damaged by insect biting into them or after germination of co-encapsulated tomato seeds (Kaya et al. 1987; Kaya and Nelsen 1985; Navon et al. 1998; Renn 1995). Moreover, the EPN in these formulations were continuously moving, which consumes energy reserves, leading to early mortality and preventing long-term storage. Chen and Glazer (2005) were the first to propose that these problems can be readily solved by inducing EPN quiescence with the addition of glycerol to the solution before encapsulation. The efficacy of this method, which we adopted for the current study, was recently validated by Kary et al. (2017, 2018), who also confirmed that EPN quiescence can be simply broken by diluting the glycerol with water. Here, we explored a novel way of making alginate beads that can contain large numbers of EPN and that can be mass-produced at low cost.

Particularly important for the success of alginate-based EPN formulations is good control over the EPN encapsulation process. Moreover, the EPN should remain healthy and be retained during storage, whereas they should be readily released when applied in soil. For this purpose, we screened a series of different concentrations of alginate and CaCl_2_·2H_2_O to determine which combination would allow the optimal EPN release within 24 h. In addition, we increased the number of encapsulated EPN to more than 4,000 per bead, instead of 100–300 as previously reported (Hiltpold et al. 2012; Kaya et al. 1987; Kaya and Nelsen 1985; Kim et al. 2015; Navon et al. 1998, 2002; Renn 1995). With these modifications, we were able to manufacture beads for long-term storage at room temperature without significant loss of EPN infectiousness and with highly effective release of thousands of EPN per bead after soil application.

Finally, we successfully tested the newly developed EPN beads under realistic field conditions against *Diabrotica virgifera virgifera*, the western corn rootworm (WCR), a univoltine chrysomelid beetle, whose larvae feed on maize roots and can cause tremendous yield losses (Chiang et al. 1980; Godfrey et al. 1993; Gray et al. 2009).

## Materials and methods

### Nematodes, insects and chemicals

Infectious juveniles of *Heterorhabditis bacteriophora* Poinar (Rhabditida: Heterorhabditidae), which have been repeatedly reported to be effective against WCR (Kurtz et al. 2009; Toepfer et al. 2005, 2008), were kindly provided by Andermatt Biocontrol AG (Grossdietwil, Switzerland) and used throughout this study. The correct taxonomic species assignment of this nematode was recently confirmed by Dr. Ricardo Machado (University of Neuchâtel, personal communication). The colony of *H. bacteriophora* was maintained on waxmoth (*Galleria mellonella* L.) larvae that were purchased from a local fishing shop as previously described (Kim et al. 2015). Newly emerged IJs from waxmoth cadavers were collected in a White trap (White 1927) and stored at a density lower than 1,000 EPN per 1-ml water in an angled-neck cell culture flask (ThermoScientific, Reinach, Switzerland), for a maximum of five days at 12 °C. IJs were examined under the microscope and used for further experiments only when all IJs were alive and active in the flask.

A strain of non-diapausing western corn rootworms (WCR) *Diabrotica virgifera virgifera* (Coleoptera: Chrysomelidae) were used for the field experiment. The strain was originally obtained from the US Department of Agriculture–Agricultural Research Service (USDA-ARS) North Central Agricultural Research Laboratory in Brookings (South Dakota, USA) and maintained in the USDA-ARS Plant Genetics Research Unit in Columbia (Missouri, USA).

Sodium alginate, Gluco® (a mixture of calcium salts of lactic and gluconic acids) and xanthan were purchased from Solé Graells (Barcelona, Spain), CaCl_2_·2H_2_O from Fluka (Buchs, Switzerland), glycerol from Sigma-Aldrich (St. Louis, MO, USA) and blue food dye from Schneitter (Neuchâtel, Switzerland). We used deionized water for all formulations.

### Evaluation of glycerol-induced quiescence of EPN in alginate capsules

Glycerol-containing Ca^2+^-alginate EPN capsules were produced at room temperature as described by Kim et al. (2015) except that in all solutions a concentration of 18% (v/v) glycerol was used. An alginate–glycerol solution (0.5% of alginate, w/v; 18% glycerol, v/v) and a mixture of Gluco® (2%, w/v), xanthan (0.4%, w/v), glycerol (18%, v/v) and blue dye (0.05%, v/v) was prepared and degassed *in vacuo*. IJs of *H. bacteriophora* were harvested from water in cell culture flasks onto a Whatman filter paper (Ø 60 mm) using a Buchner funnel and were re-suspended in the mixture of Gluco®, xanthan, glycerol and blue dye in a 15-ml Falcon tube at a concentration that ensured that each capsule would contain ca. 200 EPN. The solution was stored at 12 °C overnight and the next morning, after EPN quiescence was confirmed under an optical microscope (SZ40, Olympus, Tokyo, Japan), the EPN-containing mixture of Gluco®, xanthan, glycerol and blue dye was loaded in a 1-ml disposable syringe (Norm-Ject®, Henk-Sass Wolf GmbH, Tuttlingen, Germany) from which the barrel had been cut off, resulting in an orifice of Ø 4.5 mm. Droplets of ca. 90 μl of the mixture were dropped into 20 ml of alginate–glycerol solution in a 50-ml beaker and gently and continually stirred for 20 min. The EPN capsules that were formed were rinsed in 18% glycerol, and each stored in a Ø 60-mm disposable petri dish half immersed in 18% glycerol solution at 24 °C in the dark. As controls, we also produced ten glycerol-free EPN capsules, which were stored under the same conditions, but immersed in water without glycerol. After a week, the number of EPN that had escaped from each type of capsules was counted (*n* = 10).

### Production of EPN beads containing 18% glycerol

Glycerol-containing Ca^2+^-alginate beads were manufactured according to the modified method of Kaya and Nelsen (1985). Solutions of alginate–glycerol (0.5% sodium alginate, w/v; 18% glycerol, v/v; 0.05% blue dye, v/v; 0.075% formaldehyde, v/v) and CaCl_2_-glycerol (2% CaCl_2_·2H_2_O, w/v; 18% glycerol, v/v) were prepared and degassed *in vacuo*. IJs of *H. bacteriophora* were harvested from water in cell culture flasks onto a Whatman filter paper (Ø 60 mm) using a Buchner funnel, re-suspended in alginate–glycerol solution and stored at 12 °C overnight. The next morning, after quiescence of EPN in the alginate–glycerol solution was confirmed under an optical microscope (SZ40, Olympus, Tokyo, Japan), the EPN-alginate–glycerol solution was dripped from a 1-ml disposable syringe (Norm-Ject®, Henk-Sass Wolf GmbH, Tuttlingen, Germany) into a container with CaCl_2_-glycerol solution. Up to 100 droplets per 100 ml of CaCl_2_-glycerol solution were added, and 10 min later, Ca^2+^-alginate beads were collected. The volume of each droplet of alginate–glycerol solution was 85.3 ± 3 μl on average (*n* = 20). Different from the capsules, the alginate beads floated on the surface and did not stick to each other in the solution. Stirring of the solution during bead formation was therefore not necessary and it was possible to produce as many beads in a single container as the solution surface allowed. Glycerol-free EPN beads were produced under the same conditions.

### Comparing different concentrations of sodium alginate and CaCl_2_·2H_2_O to optimize release of EPN from the beads

EPN were harvested from water, re-suspended in a series of sodium alginate solutions at concentrations ranging from 0.5 to 2% (w/v) and dripped into a series of CaCl_2_∙2H_2_O solutions at concentrations ranging from 0.3 to 2% (w/v). Approximately 200 EPN were encapsulated in each bead. EPN beads were individually placed, half immersed in water, in a Ø 60-mm petri dish and stored at 24 °C in the dark. After a week, the number of EPN that escaped from each bead was counted under an optical microscope (*n* = 24).

### EPN escape from Ca^2+^-alginate beads containing different numbers of EPN

We also tested whether the nematodes indeed escape from the beads when their quiescence is broken by adding water. Twelve Ca^2+^-alginate beads containing on average 172, 430, 859, 1,718 and 4,295 EPN were produced under the influence of 18% glycerol as described above (named simply, 0.2 K, 0.5 K, 1 K, 2 K and 4 K EPN beads, respectively) and individually stored in a 3-ml glass vial half immersed in 300 μl of water with 18% glycerol. The vials were kept in a 500-ml plastic container at 24 °C in the dark. After a week, each bead was transferred into a 20-ml cell culture flask (ThermoScientific, Reinach, Switzerland) containing 5 ml of water in order to break quiescence. An additional week later, all the EPN that were liberated from each bead were individually counted under an optical microscope (*n* = 10 per EPN dose).

### Determination of the conversion rate of Ca^2+^ ions in a CaCl_2_∙2H_2_O solution during the production of Ca^2+^-alginate beads

Different from Ca^2+^-alginate capsules that stick together and sink to the bottom in the sodium alginate solution (Kim et al. 2015), the EPN beads described here float at the surface of CaCl_2_·2H_2_O solution and do not stick together, making the procedure highly suitable for automated mass-production. The production cycle can be continuously repeated by dropping EPN-containing sodium alginate solution into CaCl_2_·2H_2_O solution, repeatedly harvesting the beads and adding CaCl_2_·2H_2_O to the solution to compensate for the consumed Ca^2+^ ions. In order to determine how many of the Ca^2+^ ions are consumed during bead production, 100 ml of 2% CaCl_2_·2H_2_O solution was used to produce 100, 200 or 300 Ca^2+^-alginate beads in a 500-ml plastic container and the concentration of remaining Ca^2+^ ions remaining in the solution was then determined. It was possible to produce 100 beads in 100 ml of 2% CaCl_2_·2H_2_O solution in a given container, and we produced more beads in the following three rounds of 100 beads. The produced beads were removed after each round using a sieve, and the remaining CaCl_2_·2H_2_O solution was re-used for the next round. After bead production, for each round, the remaining Ca^2+^ ions were quantified using inductively coupled plasma atomic emission spectroscopy (ICP-AES; OPTIMA 3300 DV with AS-90 autosampler, Perkin Elmer, Waltham, MA, USA) at the Neuchâtel Platform of Analytical Chemistry (University of Neuchâtel, Switzerland). For the ICP-AES analyses, we used argon 5.0, nitrogen 5.0 and compressed air at 15 ml min^−1^ for plasma, 0.5 ml min^−1^ auxiliary and 0.65 ml min^−1^ for nebulizer, respectively. Samples were injected at 1.5 ml min^−1^. The concentration of Ca^2+^ ions in the samples was determined using the PlasmaCAL-SCP33MS standard solution (SCP Science, Courtaboeuf, France) prepared in 2% HNO_3_. For comparison, concentration of Ca^2+^ ions was also measured in 2% and 1.5% CaCl_2_·2H_2_O solutions. One ppm manganese solution in 2% HNO_3_ (Perkin Elmer, Waltham, MA, USA) was measured prior to each run to ensure overall suitability of the system.

### Field testing of glycerol beads to control WCR

A series of field experiments to compare the efficacy of EPN beads produced as described above were carried out in a maize field at the Bradford Research Center of University of Missouri (Columbia, MO, USA). Seeds of the WCR-susceptible maize cultivar P1420Hr CRM 114 (source: Pioneer M3PRI 11,030-N; Size: F14; DuPont Pioneer, Des Moines, IA, USA) were planted in the field on May 16th, 2014, and about 800 WCR eggs in 0.01% agar solution were applied in the soil next to each plant two weeks later. *Heterorhabditis bacteriophora* was purchased from Arbico Organics (Oro Valley, AZ, USA).

EPN beads were freshly made as described above and were applied in the soil (i) on May 22nd when the first maize seedlings started to show and WCR larvae had not yet hatched from the eggs (*early application*) or (ii) on June 11th in the middle of the growing season when WCR larvae were feeding on the roots (*late application*) or (iii) on the both dates (*early and late application*). In other plots, we used a conventional application of EPN in water solution on the same two dates of early and late applications. Empty beads, as the bead control, were also applied twice on the same dates. The blank plots received neither WCR eggs nor EPN, and the WCR control plots were treated only with WCR eggs.

Each of seven treatments was replicated 16 times in a randomized complete block design, using 16 rows of maize within which all treatments were randomly distributed. Maize plants were planted 15 cm apart from each other in a row, with 75 cm between rows. Nine plants in a row formed a plot and received the same treatment; hence, each row comprised seven plots, which were randomly assigned to each of the seven treatments. Plots were buffered with five plants between plots within the row, and the treated rows were buffered with three untreated rows of maize plants in between. On all sides, the field was surrounded by at least thirty untreated rows of maize.

**Fig. 2:**
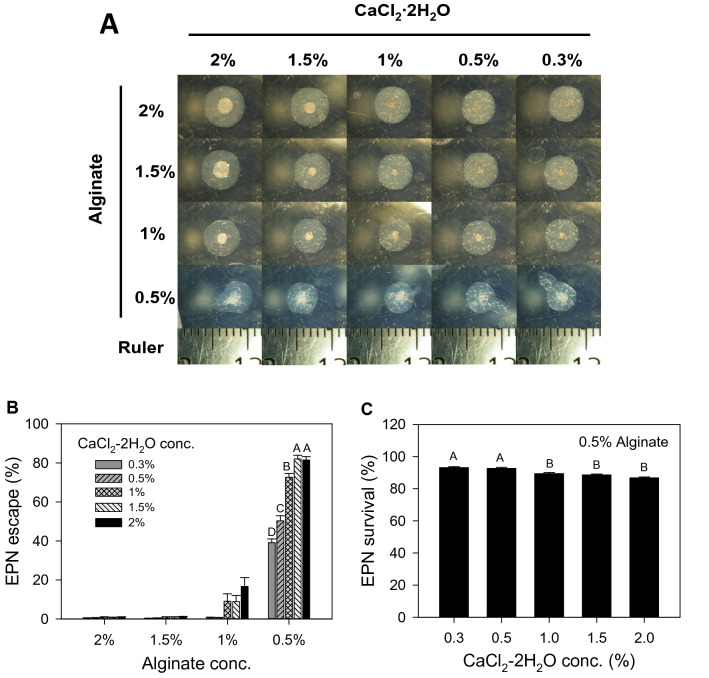
Beads made with different concentrations of sodium alginate and CaCl_2_·2H_2_O without quiescence-inducing glycerol. **a** Photographs taken right after bead production from different concentration combinations. Notice the different accumulations of EPN at the center of the beads and the different number of EPN that were caught in the outer part. The smaller markings on the ruler are 1 mm apart. **b **The number of EPN that escaped from each bead was counted a week after the beads were formed and the data from beads formed with 0.5% alginate concentration are shown in the graph (mean ± SE; Kruskal–Wallis test followed by post hoc Tukey’s test; *χ*^2^ = 89.9519, *p* < 0.0001, *df* = 4, *n* = 24, *N* = 120) and **c** the number of dead EPN from the beads formed with 0.5% alginate concentration was also counted to obtain survivorship values (mean ± SE; ANOVA test followed by post hoc Tukey’s test; *F*_4,115_ = 15.5, *p* < 0.0001, *df* = 4, *n* = 24, *N* = 120). Bars represent SEM, and capital letters indicate statistical differences between treatments

To apply each treatment, two holes of about 1 cm in diameter and 20 cm deep were made about 15 cm away from a plant and on both sides of and perpendicularly to the plant row. For the application of the EPN solution, the solution was prepared fresh by adding EPN to water at a concentration of 1,000 ml^−1^, 15 ml of which was applied into each of two holes with a pipette (representing 30,000 EPN per plant). During application, the solution was agitated frequently to prevent EPN settlement at the bottom of the container. For the bead application, 4 K EPN beads, as well as the control beads were produced with 18% glycerol as described above a day before application, and ten beads were dropped into the two holes, five beads per hole (assuming an escape rate of 80%, Fig. 2, this represented about 32,000 per plant). The holes were re-filled with soil after application of the solution or beads.

Maize plants from all plots were uprooted on July 10^th^. Soil on roots was washed off with water, and root damage by WCR was evaluated according to the 0–3 node injury scale (Oleson et al. 2005).

### Statistical analyses

All the data presented in this manuscript were tested with SAS (SAS 9.3, SAS Inc.) for normality and equal variances, and means were tested for significant differences using one-way ANOVA test or Kruskal–Wallis test, accordingly. Multiple comparisons of data were conducted using post hoc Tukey’s test when *p* < 0.05.

## Results

### Glycerol-induced quiescence of EPN

After overnight treatment in an 18% glycerol solution, all EPN were found dormant the next morning. When encapsulated as described by Kim et al. (2015) and stored partially immersed in a 18% glycerol solution at room temperature for a week, not a single EPN was found outside of the capsules. Yet, when the glycerol was diluted by adding water almost all EPN became active and left the capsules. However, from capsules made under the same conditions but without 18% glycerol, a significantly number of EPN were found to escape prematurely (37.7 ± 13.2% vs. 0%, mean ± SE, Kruskal–Wallis test; *χ*^2^ = 6.1688, *p* = 0.013, *df* = 1, *n* = 10, *N* = 20). When we embedded the EPN in solid alginate beads with 18% glycerol, the EPN were also completely retained and quiescence was maintained until the glycerol was diluted with an excess of water.

### Escape of EPN from Ca^2+^-alginate capsules and beads

Alginate solutions of concentrations higher than 2% were too thick to handle. Also, beads were not properly formed when the concentrations of sodium alginate and CaCl_2_∙2H_2_O solutions were below 0.5% and 0.3%, respectively. We therefore manufactured alginate beads (with about 200 EPN) from solutions with concentrations of sodium alginate and CaCl_2_·2H_2_O ranging from 0.5 to 2.0% and 0.3 to 2.0%, respectively, initially without glycerol. Beads formed from 0.5% of sodium alginate solution in CaCl_2_·2H_2_O solution of any concentrations between 0.3 and 2.0% were not spherical as shown in Fig. 2, but still sufficiently firm for easy handling and became almost spherical after more practice in making them. We did not use CaCl_2_·2H_2_O solution over 2% to avoid salinity stress. Photographs of EPN beads were taken right after production, and a week later, the number of EPN that had escaped from each bead was counted (Fig. 2).

The photographs in Fig. 2 show how the EPN behave during bead formation. After a droplet of sodium alginate solution is dropped into the CaCl_2_·2H_2_O solution, cross-linking of alginate polymers by Ca^2+^ ions starts right away at the boundary of the two solutions and the Ca^2+^-alginate bead matrix grows inward. In general, a small number of EPN were found trapped in the matrix, but the majority moved to the center. Judging from the decreasing number of EPN in the center and the increasing number embedded within the bead matrix at the higher concentrations of CaCl_2_·2H_2_O, the Ca^2+^-alginate cross-linking occurred more rapidly when the concentrations of CaCl_2_·2H_2_O were higher (Fig. 2a).

When EPN beads were formed with 1.5% or 2.0% sodium alginate solutions, less than 1% of the encapsulated EPN escaped from the beads, irrespective of the concentrations of CaCl_2_·2H_2_O (Fig. 2b). Escape rates were significantly higher when beads were formed by dropping droplets of 0.5% sodium alginate solution into a CaCl_2_·2H_2_O solution, and the highest EPN escape rate of about 82% was achieved when EPN beads were produced with a 0.5% sodium alginate solution combined with a 1.5% or 2.0% CaCl_2_·2H_2_O solution (Kruskal–Wallis test followed by post hoc Tukey’s test; *χ*^2^ = 89.9519, *p* < 0.0001, *df* = 4, *n* = 24, *N* = 120) (Fig. 2b). Survival of EPN from beads made with a 0.5% sodium alginate solution decreased with increased concentrations of CaCl_2_·2H_2_O, but remained still very high, ranging from 86.7 to 93.0% (ANOVA test followed by post hoc Tukey’s test; *F*_4,115_ = 15.5, *p* < 0.0001, *n* = 24, *N* = 120) (Fig. 2c). Because calcium ions in the CaCl_2_·2H_2_O solution are continuously consumed when cross-linking alginate polymers, we selected 2.0% instead of 1.5% of CaCl_2_∙2H_2_O for the following experiments to have more Ca^2+^ ions available for cross-linking.

### Escape rates from Ca^2+^-alginate beads with different numbers of EPN

About 82% of the EPN escaped from beads that had been manufactured without glycerol, as described above. In contrast, when we added 18% glycerol to similar beads, only half of the EPN (48.1 ± 1.9%, mean ± SE, *n* = 24) managed to escape after the beads were placed in water, prompting us to try to find a way to enhance the escape rate. We first tested if this could be achieved with larger numbers of EPN per bead, expecting that the higher numbers of EPN would facilitate movement through the matrix.

**Fig. 3:**
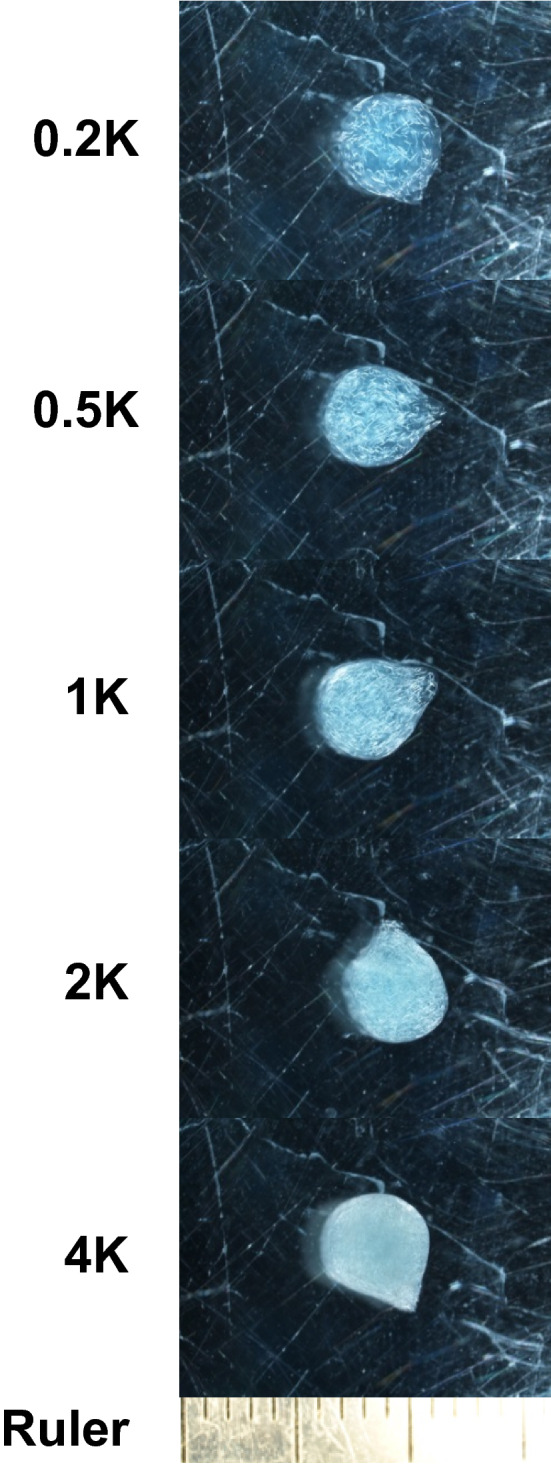
Beads with approximately 200, 500, 1,000, 2,000 and 4,000 EPN under the influence of glycerol-induced quiescence (see Table 1for details). EPN beads were formed with 0.5% sodium alginate and 2% CaCl_2_∙2H_2_O solutions as described in the text. The smaller markings on the ruler are 1 mm apart

We prepared 0.5% alginate solutions containing 18% glycerol, 0.05% blue dye, 0.075% formaldehyde with 200, 500, 1000, 2000 or 4000 EPN per 100 μl. There was no significant difference in droplet size of solutions containing different numbers of EPN (ANOVA; *F*_4,45_ = 1.957, *p* = 0.117, *n* = 10, *N* = 50), and the mean volume of a droplet of all the EPN-alginate solutions was 85.9 ± 0.7 μl (mean ± SE). Thus, the number of EPN in the beads manufactured from these solutions was estimated to be 172, 430, 859, 1718 and 4295, which we simply refer to as 0.2 K, 0.5 K, 1 K, 2 K and 4 K EPN beads, respectively. Only 0.17, 0.37, 0.33, 0.51 and 0.52% of the EPN escaped on average from these beads, respectively, during the 7 days that the beads were stored in the presence of glycerol. The 4 K EPN beads were completely filled with EPN, but the beads still maintained a solid and round form (Fig. 3).

After 7 days of storage at room temperature, glycerol-induced quiescence was broken by adding water, allowing EPN escape for the next 7 days. Then, EPN that had escaped out of each bead were counted as dead or alive under a microscope (Table 1). Almost 80% of the EPN escaped from the 0.2 K EPN beads, and the escape rate increased with the number of EPN per bead, reaching almost 90% for the 4 K EPN beads. Moreover, survival rate was very high (98.1–99.5%). Based on these results, 4 K EPN beads produced with 0.5% alginate and 2% CaCl_2_·2H_2_O solutions with the addition of 18% glycerol were used for the field experiment to test their efficacy in controlling WCR (Table 2).

**Table 1:** The number of EPN that were incorporated into and emerged from glycerol-Ca^2+^-alginate beads and the survival rate of EPN (*n* = 10)

EPN beads	Number of EPN per bead		EPN release rate (%)	EPN survival rate (%)***
	Incorporated*	Released** (Mean ± SE)	(Mean ± SE)	(Mean ± SE)
0.2 K	172	134.7 ± 3.1	78.3 ± 1.8	99.8 ± 0.1
0.5 K	430	265.4 ± 9.9	61.7 ± 2.3	99.8 ± 0.1
1 K	859	607.9 ± 18.5	70.8 ± 2.2	99.4 ± 0.2
2 K	1,718	1,483.8 ± 25.4	86.4 ± 1.5	98.6 ± 0.3
4 K	4,295	3,758.1 ± 91.4	87.5 ± 2.1	98.1 ± 0.4

*The number of incorporated EPN per bead estimated from the mean volume of a droplet of EPN-containing glycerol-alginate solution (85.9 ± 0.7 μl, mean ± SE, *n* = 50). EPN-containing glycerol-alginate solution was prepared to have approximately 200, 500, 1,000, 2,000 and 4,000 EPN per 100 μl and simply named 0.2 K, 0.5 K, 1 K, 2 K and 4 K EPN beads, respectively. **After 7 days of storage of EPN beads under the influence of 18% of glycerol, glycerol-induced EPN quiescence was broken by diluting out glycerol of beads with excess of water and the number of released EPN was individually counted. ***The number of live EPN divided by the number of all the escaped EPN

**Table 2:** Test groups assigned in the field experiment

Test groups	Treatments		WCR eggs	EPN	Beads
No WCR / No EPN	Blank		−	−	−
WCR only	WCR eggs only		+	−	−
Empty beads	Bead control: Ca^2+^-alginate beads without EPN		+	−	+
EPN water	Application of EPN with water		+	+	−
EPN beads	Early	Application of 4 K EPN beads before WCR larvae hatched	+	+	+
	Late	Application of 4 K EPN beads during the active root herbivory by WCR	+	+	+
	E + L	Two-time application of 4 K EPN beads: Early + Late	+	+	+

### Expenditure of Ca^2+^ ions in production of Ca^2+^-alginate beads

Different from Ca^2+^-alginate capsules (Hiltpold et al. 2012; Kim et al. 2015), beads can be produced as many as the surface area of the formulation container allows because the Ca^2+^-alginate matrix grows inward, and therefore, Ca^2+^-alginate beads do not stick to each other. This should simplify mass-production as long as sufficient Ca^2+^ ions are added to the solution. We determined how much Ca^2+^ ions participated in the formation of 100 Ca^2+^-alginate beads and consumed from 100 ml of 2% CaCl_2_·2H_2_O solution using inductively coupled plasma atomic emission spectroscopy (ICP-AES). The concentration of Ca^2+^ ions in 1.5% and 2% CaCl_2_∙2H_2_O solutions was 3.78 mg ml^−1^ and 5.21 mg ml^−1^, respectively. After the first production of 100 Ca^2+^-alginate beads in the 2% CaCl_2_∙2H_2_O solution, the concentration of Ca^2+^ ions decreased to 4.63 mg ml^−1^. After the second production of 100 beads, the concentration of Ca^2+^ ions decreased to 4.08 mg ml^−1^, and after the third production of 100 beads, the concentration of Ca^2+^ ions decreased to 3.68 mg ml^−1^. This final concentration is almost the same as the starting concentration of Ca^2+^ ions in a 1.5% CaCl_2_·2H_2_O solution (3.78 mg ml^−1^). Based on these results, we calculated that about 1.13 mg ml^−1^ of Ca^2+^ ions were consumed per 100 Ca^2+^-alginate beads.

### Efficacy of 4 K EPN glycerol beads for the control of WCR in the field

To test the efficacy of the novel bead formulation under realistic conditions, we carried out a field experiment with 4 K EPN beads and tested how their application may reduce root damage by WCR larvae in a maize field in Missouri, USA. Because it took 8–16 h for EPN to start waking up from the glycerol-induced quiescence (Fig. S1), the beads were already transported in water to the field where they were then buried in the soil.

**Fig. 4:**
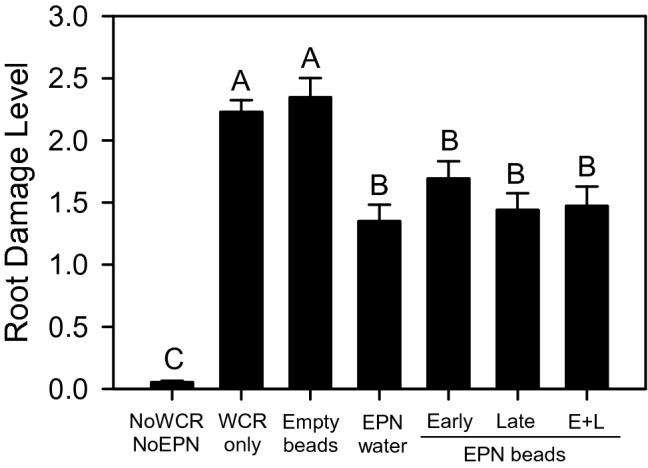
Efficacy of EPN in protecting maize roots from WCR larvae in a field trial. Control plants (NoWCR, NoEPN) suffered significantly less damage than any other of the treatments. Plots with insect infestation but no EPN (WCR only and Empty beads) had the most damage, whereas EPN application (EPN water, Early/Late/Early + Late (E + L) EPN beads) suffered significantly less damage, whether the EPN were sprayed or applied in 4 K beads. EPN beads were formed with 0.5% sodium alginate and 2% CaCl_2_·2H_2_O solutions as described in the text. Bars represent SEM, and capital letters indicate statistical differences between treatments

The maize plants from plots that received no WCR eggs and no EPN application showed minimal root damage (Fig. 4). The plants in plots that were treated with only WCR eggs and in plots treated with WCR eggs plus empty beads showed the greatest level of root damage. In the remaining plots, where EPN were applied, root damage was significantly reduced (Fig. 4). No differences between the four different types of EPN applications were found, and 4 K EPN beads were as efficient in reducing maize root damage as EPN that were applied in water. There was also no difference in effectiveness between early application and late application or combined early and late applications (maize root damage, mean ± SE: Kruskal–Wallis test followed by post hoc Tukey’s test with *p* < 0.05; *χ*^2^ = 66.0252, *df* = 6, *p* < 0.0001, *n* = 16; *N* = 112) (Fig. 4).

## Discussion

In this study, we developed mass-producible Ca^2+^-alginate EPN beads that can house and liberate as many as 4,000 EPN per 90-μl bead. EPN incorporated in these beads were stored without refrigeration in a state of glycerol-induced quiescence. It is known that that EPN can be conserved like this for at least 6 months without affecting infectivity (Chen and Glazer 2005). We also show in a field experiment that the application of the 4 K EPN beads into the soil around maize plants was as efficient in protecting maize roots against rootworms as EPN applied in water.

In a previous study (Kim et al. 2015), we focused on the chemical and physical properties of Ca^2+^-alginate capsules (not beads), seeking conditions under which EPN are best retained in the formulation. We had improved the retainment of EPN inside these capsules by about 40%. However, for an effective control of root pests, the release of EPN out of the formulation into the soil is as important as retaining them inside during storage. The initial capsule formulation was also problematic because it relied on dripping droplets of sodium alginate solution into CaCl_2_·2H_2_O solution, whereby the capsule shell grows outward and the growing capsules readily stick to each other (Kim et al. 2015). Moreover, the concentration of alginate polymer in the sodium alginate solution decreases after production of a single capsule, requiring that it is continuously replaced with fresh alginate solution, which is expensive and time consuming.

To develop a formulation with prolonged retainment and controlled release of EPN, we adopted two new strategies. Firstly, we used glycerol to induce EPN quiescence. If they are not in a state of quiescence, EPN will continuously try to wiggle out of the capsule, depleting their energy resources and losing infectivity. In a dormant state, EPN will stay vigorous and infectious for a considerable time until quiescence is broken (Hiltpold et al. 2015; Jaffuel et al. 2015). Secondly, we used concentrations of sodium alginate and CaCl_2_·2H_2_O that produce beads from which EPN can readily escape when quiescence is broken by adding water. After this, it takes about 8–16 h for EPN to recover. For the field experiment, we therefore placed 4 K EPN beads in water just before going out to the field. In the field, 4 K EPN beads were found to be as efficient in reducing WCR herbivory as EPN that were applied in water (Fig. 4).

The first-reported Ca^2+^-alginate EPN beads were produced with 2% sodium alginate and 100 mM of CaCl_2_·2H_2_O solutions (Kaya et al. 1987; Kaya and Nelsen 1985). According to our screening results, such a formulation would release only a limited number of EPN, whereas our beads with 0.5% sodium alginate and 2% CaCl_2_·2H_2_O (27 mM of Ca^2+^ ions) allow the release of most of the embedded EPN. In previous studies, spontaneous escape of EPN out of Ca^2+^-alginate formulations was not considered (Hiltpold et al. 2012; Kim et al. 2015; Navon et al. 1998, 2002; Renn 1995). Instead, the authors adopted different strategies to liberate EPN. Kaya et al. (1987) used a citrate solution to dissolve Ca^2+^-alginate matrix, which could be useful to liberate EPN after storage in Ca^2+^-alginate beads (Chen and Glazer 2005), but not practical for the field application (Grewal 2002). Kaya et al. (1987) also introduced the elegant strategy of encapsulating a crop seed in each bead. The germinating seeds open up the beads, allowing EPN to be liberated. This approach, however, exposes the seeds to humidity, rapidly inducing germination, and thus making the beads not suitable for long-term storage. Lastly, alginate gels or capsules have also been spiked with feeding stimulants or attractants for target insects, such as yeast extract, sugar or fatty acids (Hiltpold et al. 2012; Navon et al. 1998, 2002). Navon et al. (1998; 2002) used a gel disk formulation containing yeast extract, which they applied to the canopy of cotton plants infested with caterpillars. Hiltpold et al. (2012) incorporated several known attractants of WCR larvae into alginate capsules, which enhanced the effectiveness of the capsules in the field. Compared to these formulations, our beads should allow for long-term storage, for several months (Chen and Glazer 2005), without affecting EPN infectivity and they can be “planted” along with crop seeds, which would be far less labor intensive than conventional EPN application methods.

Also, the bead formulation allows for the encapsulation of large numbers of EPN. Quiescence induction was found to be essential to prevent the EPN from moving to the center during bead formation. When a droplet of sodium alginate solution is dropped into CaCl_2_∙2H_2_O solution, a Ca^2+^-alginate network starts to form at the boundary of the two solutions and this matrix grows inward. Non-dormant EPN move along to the center, but as shown in Figs. 2 and 3, with quiescence induction, this movement could be prevented. We successfully encapsulated as many as 4,000 EPN per 5-mm diameter bead (Fig. 3). Interestingly, with these high numbers, EPN release rate was highest; after submergence in water, almost 90% made their way out of the beads. The better release from the most densely packed beads may be due to a looser matrix structure because a lower ratio of alginate molecules participating in the formation of the Ca^2+^-alginate network. With more EPN per bead, only a small number of EPN beads are needed to effectively control root pests within each root system.

Application of 4 K EPN beads only once early in the growing season was as efficient in protecting maize roots from WCR damage as the double application of free EPN in water. These results are of great practical importance because they imply that a one-time application of 4 K EPN beads along with planting maize seeds may be enough to protect maize roots from WCR damage throughout the season.

Georgis et al. (2006) list a number of requirements that are important for commercialization of EPN. These include good control efficacy against the target pest under the field conditions, low production cost, ease of handling and transportation, and long shelf-life. Our version of Ca^2+^-alginate beads meets these criteria, but with a couple of minor issues still to be considered. First, different species and strains of EPN show different efficacies in killing a given insect pest, which may indicate limitations to their respective host range (Peters 1996). We used *H. bacteriophora* that has been reported to be effective against WCR larvae (Kurtz et al. 2009; Toepfer et al. 2005, 2008). Other species may require fine adjustments to the conditions required for successful encapsulation. Second, in the field study carried out in this study, the beads were transferred from the laboratory to the maize field in water to quickly break glycerol-induced quiescence. For future application, it may be more practical to directly bury the beads. In that case, the breaking of quiescence will mainly depend on soil water content. Ideally, the formulation would be adjusted to ensure a continuous release of EPN into the rhizosphere beyond the moment WCR larvae hatch and start to inflict damage to the roots. Irrigation timing may be adapted to facilitate EPN release at the right moment. On the other hand, low soil water content may facilitate a continuous release of EPN into the rhizosphere throughout growing season.

In summary, in this study we present a new formulation of Ca^2+^-alginate beads that allow complete retainment of several thousands of EPN per 4–5-mm diameter bead. During storage, glycerol-induced quiescence keeps the EPN inside the beads. Quiescence can be readily broken by adding water just before soil application. In a field trial, the beads were as effective in protecting maize roots from WCR larvae as EPN that were applied in a water solution. Different from previously reported alginate-based EPN formulations, the Ca^2+^-alginate EPN beads are expected to be suitable for mass-production and for long-term storage (at least for 6 months), and can, with some modification, be directly applied with the seeds during sowing. Further investigation into the dynamics of EPN release from the beads under a variety of growing conditions is warranted.

## Supplementary information

Below is the link to the electronic supplementary material.

The number of EPN that emerged from 4K EPN Ca^+^-alginate beads over time (within 16 h) after the beads were placed in water (mean ± SE, Kruskal-Wallis test followed by post-hoc Tukey’s test;* χ*^2^= 38.4201,* p*<0.0001, df = 3,* n* = 12,* N* = 48). (PPTX 44 KB)

## Data Availability

All relevant data are included in the manuscript.
