# Supplementary figures and images for: Calcium-alginate beads as a formulation for the application of entomopathogenic nematodes to control rootworms
Source: J Pest Sci (2004). 2021 Feb 26;94(4):1197–208. doi: 10.1007/s10340-021-01349-4 (PMC8550308; doi:10.1007/s10340-021-01349-4)

## Slide 1
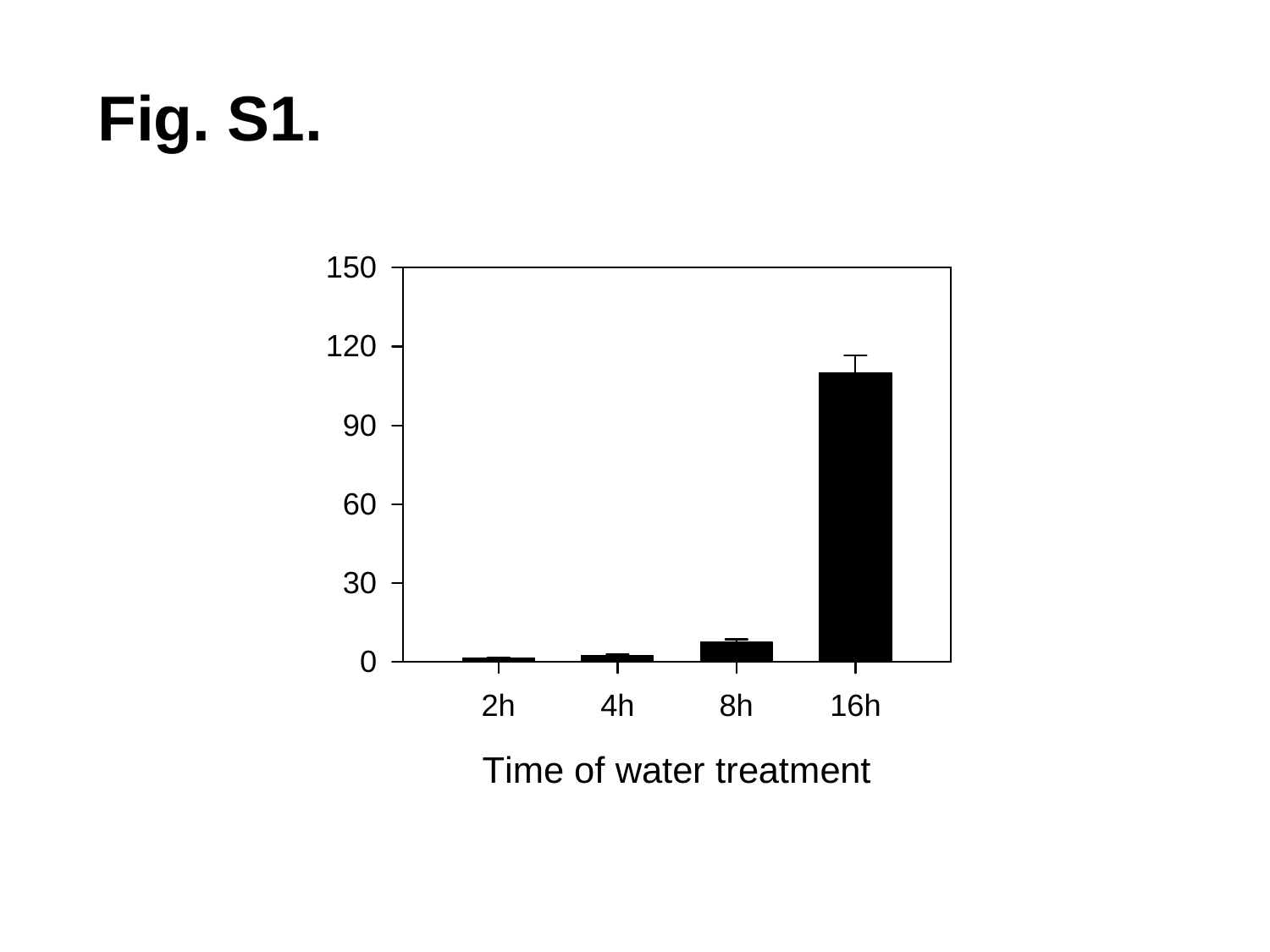

Fig. S1.

Supplement: Supplementary file 1 — The number of EPN that emerged from 4K EPN Ca+-alginate beads over time (within 16 h) after the beads were placed in water (mean ± SE, Kruskal-Wallis test followed by post-hoc Tukey’s test; χ2= 38.4201, p<0.0001, df = 3, n = 12, N = 48). (PPTX 44 KB) [file 10340_2021_1349_MOESM1_ESM.pptx]
